# Antigen I/II Participates in the Interactions of *Streptococcus suis* Serotype 9 With Phagocytes and the Development of Systemic Disease

**DOI:** 10.3389/fcimb.2019.00124

**Published:** 2019-04-24

**Authors:** Jean-Philippe Auger, Anaïs-Christelle Boa, Mariela Segura, Marcelo Gottschalk

**Affiliations:** Research Group on Infectious Diseases in Production Animals (GREMIP) and Swine and Poultry Infectious Disease Research Center (CRIPA), Faculty of Veterinary Medicine, University of Montreal, Saint-Hyacinthe, QC, Canada

**Keywords:** *Streptococcus suis* serotype 9, antigen I/II, virulence, systemic infection, macrophages, dendritic cells

## Abstract

*Streptococcus suis* is an important porcine bacterial pathogen and a zoonotic agent causing a variety of pathologies including sudden death, septic shock, and meningitis. Though serotype 2 is the most studied serotype due to its presence worldwide, serotype 9 is responsible for the greatest number of porcine cases in Spain, the Netherlands, and Germany. Regardless of its increasing importance, very few studies have investigated *S. suis* serotype 9 virulence factors and pathogenesis. Antigens I/II (AgI/II) are multimodal adhesion proteins implicated in host respiratory tract and oral cavity persistence of various pathogenic human streptococci. It was recently demonstrated that AgI/II is involved in various bacterial functions for serotype 9, participating in the initial steps of the pathogenesis of the infection. However, its contribution to the systemic infection remains unknown. As such, we evaluated herein the role of the *S. suis* serotype 9 AgI/II in the interactions with phagocytes and the development of systemic disease in a mouse model of infection. Results demonstrated that the presence of AgI/II is important for the development of clinical systemic disease by promoting bacterial survival in blood possibly due to its effect on *S. suis* phagocytosis, as shown with macrophages and dendritic cells. Furthermore, AgI/II directly participates in dendritic cell activation and pro-inflammatory mediator production following recognition by the Toll-like receptor pathway, which may contribute to the exacerbated systemic inflammation responsible for host death. Taken together, this study demonstrates that the *S. suis* serotype 9 AgI/II is important for virulence during systemic infection and development of disease. In fact, this is the first study to describe a role of an AgI/II family member in systemic bacterial disease.

## Introduction

*Streptococcus suis* is a bacterial pathogen of post-weaning piglets responsible for important economic losses, with sudden death, meningitis, and arthritis being the most frequent clinical manifestations (Gottschalk et al., 2010). Moreover, *S. suis* is a zoonotic agent responsible for meningitis and septic shock in humans (Wertheim et al., 2009). Of the different described serotypes, serotype 2 is the most widespread and virulent (Goyette-Desjardins et al., 2014). In recent years, however, serotype 9 has emerged in Europe and is presently responsible for the greatest number of porcine cases of *S. suis* infection in Spain, the Netherlands, and Germany (Goyette-Desjardins et al., 2014). Furthermore, its prevalence in China (Zhu et al., 2011) and Canada (Gottschalk and Lacouture, 2015) has increased, with the first human case of *S. suis* serotype 9 infection reported in 2015 (Kerdsin et al., 2015). Regardless of its increasing importance, however, very few studies have investigated serotype 9 virulence factors and pathogenesis, with our understanding of the *S. suis* pathogenesis mostly based on serotype 2 studies (Wertheim et al., 2009; Fittipaldi et al., 2012; Segura et al., 2016). Nevertheless, certain factors have been proposed to be involved in serotype 9 survival, fitness, and persistence, as well as in host immune response interference, namely the LysM domain surface protein (Wu et al., 2016), a 5′-nucleotidase (Dai et al., 2018), the XRE family transcription regulator SrtR (Hu et al., 2018), and the type VII secretion system putative substrate EsxA (Lai et al., 2017).

Following colonization of the upper respiratory tract or tonsils of pigs, virulent strains may reach the bloodstream after breaching the mucosal epithelium (Segura et al., 2016). Moreover, *S. suis* is also able to interact with porcine intestinal epithelial cells (Ferrando et al., 2015). Similarly, infection in humans occurs via skin wounds or at the intestinal interface following ingestion of raw or undercooked infected pork products (Ferrando et al., 2015; Segura et al., 2016). In the bloodstream, *S. suis* resists killing by phagocytes, which allows bacterial multiplication resulting in bacteremia, organ dissemination, and development of systemic infection (Fittipaldi et al., 2012). Moreover, *S. suis* activates blood and tissue-resident innate immune cells, including macrophages and dendritic cells (DCs), which participate in the massive release of pro-inflammatory mediators. This response results in exacerbated inflammation responsible for sepsis leading to sudden death in pigs and septic shock in humans (Gottschalk et al., 2010). If untreated, *S. suis*-induced systemic inflammation may end in host death (Gottschalk et al., 2010). However, the precise mechanisms and virulence factors involved are poorly understood.

Antigens I/II (AgI/II) have been extensively described in oral and invasive pathogenic streptococci, including *Streptococcus mutans, Streptococcus gordonii*, Group A *Streptococcus* (GAS; *Streptococcus pyogenes*), and Group B *Streptococcus* (GBS; *Streptococcus agalactiae*) (Brady et al., 2010). These multimodal adhesion proteins are implicated in host respiratory tract and oral cavity persistence and in dissemination (Brady et al., 2010). Importantly, they have been described to possess a wide variety of functions ranging from self-aggregation, aggregation to soluble and surface-immobilized components, biofilm formation, and cell adhesion (Brady et al., 2010). We recently demonstrated that not only is AgI/II present in *S. suis* serotypes 2 and 9, but that its role is serotype-specific (Chuzeville et al., 2017). Indeed, while AgI/II is involved in the above mentioned functions for serotype 9, very little functions are associated with its presence in serotype 2 (Chuzeville et al., 2017). As such, AgI/II participates in the initial steps of the *S. suis* serotype 9 pathogenesis, more specifically in colonization, persistence, and carriage in the upper respiratory tract and tonsils of pigs (Chuzeville et al., 2017).

It must be noted that studies on the role of streptococcal AgI/II have been generally limited to respiratory tract colonization and persistence. In fact, little information is available regarding other functions and/or environments. Yet, the *S. suis* serotype 9 AgI/II might also be involved elsewhere, as shown for the GAS AgI/II protein (termed AspA), that protects bacteria against phagocytosis and killing by macrophages and neutrophils (Franklin et al., 2013). In fact, such functions could be important for the *S. suis* pathogenesis since they are required for bacterial survival and dissemination and consequent development of systemic disease (Fittipaldi et al., 2012).

Consequently, we evaluated the role of the *S. suis* serotype 9 AgI/II in the interactions with phagocytes and the development of systemic disease in a mouse model of infection. Results demonstrated that AgI/II promotes bacterial survival in blood and tissues due to its effect on *S. suis* phagocytosis. Furthermore, it also participates in innate immune cell activation and pro-inflammatory mediator production, which contribute to the exacerbated systemic inflammation and host death.

## Materials and Methods

### Bacterial Strains and Growth Conditions

The well-encapsulated wild-type *S. suis* serotype 9 1135776 strain, isolated from a diseased pig in Canada and belonging to sequence type 788 (Zheng et al., 2018), and its previously constructed isogenic AgI/II-deficient mutant (Δ*agI/II*) and complemented strain (CΔ*agI/II*) (Chuzeville et al., 2017), were used. Strains were cultured in Todd Hewitt broth (THB; Becton Dickinson, Mississauga, ON, Canada). For *in vitro* cell culture and whole blood bactericidal assays, bacteria were prepared as previously described (Lecours et al., 2011) and resuspended in cell culture medium. For experimental infections, early stationary phase bacteria were washed twice in phosphate-buffered saline, pH 7.4 (PBS), and resuspended in THB (Dominguez-Punaro et al., 2007, 2008; Auger et al., 2016). Bacterial cultures were appropriately diluted and plated on THB agar (THA) to accurately determine bacterial concentrations. For the complemented strain, spectinomycin (Sigma-Aldrich, Oakville, ON, Canada) was added at a concentration of 500 μg/mL.

### Purification of Recombinant Antigen I/II

Recombinant AgI/II (rAgI/II) was expressed and purified as previously described (Chuzeville et al., 2017). Briefly, the serotype 9 *agI/II* gene was cloned into the pET151 expression vector (Invitrogen, Burlington, ON, Canada) according to the manufacturer's instructions. Protein synthesis was induced using 0.5 mM of isopropyl β-d-1-thiogalactopyranoside (Sigma-Aldrich) and cells lysed using lysozyme (Sigma-Aldrich) and sonication. The resulting recombinant His-tagged AgI/II (rAgI/II) was purified by affinity chromatography using the His-Bind Resin Chromatography Kit (Novagen, Madison, WI, USA,) according to manufacturer's instructions. Protein purity was evaluated by sodium dodecyl sulfate–polyacrylamide gel electrophoresis following dialysis. Protein concentration was determined using the Pierce Bicinchoninic Acid (BCA) Protein Assay Kit (Thermo Scientific, Waltham, MA, USA).

### *Streptococcus suis* Serotype 9 Systemic Infection Mouse Virulence Models

Six-week-old male and female C57BL/6 mice (Jackson Research Laboratories, Bar Harbor, ME, USA) were used throughout this study. Mice were acclimatized to standard laboratory conditions with unlimited access to water and rodent chow (Dominguez-Punaro et al., 2008; Auger et al., 2016). These studies were carried out in strict accordance with the recommendations of and approved by the University of Montreal Animal Welfare Committee guidelines and policies, including euthanasia to minimize animal suffering, applied throughout this study when animals were seriously affected since mortality was not an endpoint measurement. After standardization trials with the wild-type strain to determine the ideal dose, *S. suis* strains were inoculated at a final dose of 1 × 10^7^ colony forming units (CFU) to groups of 10 mice by either the intraperitoneal or intravenous (caudal vein) route for survival and blood bacterial burden evaluation. Mice were monitored at least three times daily until 72 h post-infection and twice thereafter until 14 days post-infection. Blood bacterial burden of surviving mice was assessed 24 h post-infection by collecting blood from the caudal vein, appropriately diluting, and plating on THA or THA containing spectinomycin for the complemented strain, as described above. Blood bacterial burden was also measured upon euthanasia.

### Measurement of Plasma (Systemic) Pro-inflammatory Mediators

In addition, eight mice per group were intraperitoneally mock-infected (THB) or infected with 1 × 10^7^ CFU of the *S. suis* strains and blood collected 12 h post-infection by intracardiac puncture following euthanasia and anti-coagulated with EDTA (Sigma-Aldrich) as previously described (Lachance et al., 2013; Auger et al., 2016). Plasma supernatants were collected following centrifugation at 10,000 × *g* for 10 min at 4°C and stored at −80°C. The 12 h post-infection time point was selected to obtain maximal pro-inflammatory mediator production in the absence of significant mouse mortality as determined in a preliminary study (data not shown). Plasmatic concentrations of interleukin (IL)-6, IL-12p70, interferon (IFN)-γ, C-C motif chemokine ligand (CCL) 2, CCL3, CCL4, C-X-C motif chemokine ligand (CXCL) 1, and CXCL2 were measured using a custom-made cytokine Bio-Plex Pro™ assay (Bio-Rad, Hercules, CA, USA) according to the manufacturer's instructions. Since data with serotype 9 is limited, mediators were selected based on serotype 2 studies and represent the most important pro-inflammatory cytokines and chemokines secreted (Dominguez-Punaro et al., 2007, 2008; Auger et al., 2016, 2017). Acquisition was performed on the MAGPIX platform (Luminex®) and data analyzed using the Bio-Plex Manager 6.1 software (Bio-Rad).

### Peritoneal Macrophage Isolation

Resident peritoneal macrophages were isolated from C57BL/6 mice as previously described (Segura et al., 1998) with some modifications. Cells were recovered by washing the peritoneal cavity with cold PBS without prior elicitation, pooled, and resuspended in Dulbecco's Modified Eagle's Medium (Gibco, Burlington, ON, Canada) supplemented with 10% heat-inactivated fetal bovine serum (Gibco) and plated at 1 × 10^5^ cells/mL. Peritoneal macrophages were allowed to adhere for 1 h at 37°C with 5% CO_2_ and then washed twice with warm PBS to remove non-adherent cells prior to infection.

### Generation of Bone Marrow-Derived Dendritic Cells

The femur and tibia of wild-type, MyD88^−/−^ (B6.129P2(SJL)-*MyD88*^*tm*1.*Defr*^/J), TLR2^−/−^ (B6.129-*Tlr2*^*tmKir*^/J), or TLR4^−/−^ (B6.B10ScN-*Tlr4*^*lps*−*del*^/JthJ) C57BL/6 mice were used to generate bone marrow-derived DCs, as previously described (Lecours et al., 2011). Briefly, hematopoietic bone marrow stem cells were cultured in RPMI-1640 medium (Gibco) supplemented with 5% heat-inactivated fetal bovine serum, 10 mM HEPES (Gibco), 2 mM L-glutamine (Gibco), and 50 μM 2-mercaptoethanol (Gibco). Complete medium was complemented with 20% granulocyte-macrophage colony-stimulating factor from mouse-transfected Ag8653 cells (Segura et al., 2007). Prior to infection, cells were plated at 1 × 10^6^ cells/mL. Cell purity was determined to be at least 85% CD11c^+^ by flow cytometry.

### Internalization and Intracellular Survival Assays

Bacteria were pre-opsonized with either 20% complete or heat-inactivated normal C57BL/6 mouse serum in PBS for 30 min at 37°C with shaking as previously described (Lecours et al., 2011). Cells were infected with *S. suis* strains at optimal conditions in culture medium (1 × 10^7^ CFU for peritoneal macrophages and 1 × 10^8^ CFU for DCs; multiplicity of infection [MOI] = 100) and phagocytosis was left to proceed for 1 h at 37°C with 5% CO_2_. After incubation, penicillin G (5 mg/mL; Sigma-Aldrich) and gentamicin (100 mg/mL; Gibco) were directly added to the wells for 1 h to kill extracellular bacteria. Supernatant controls were taken in every test to confirm that extracellular bacteria were efficiently killed by the antibiotics. After antibiotic treatment, cells were washed three times and lysed using water. The number of CFU recovered was determined by plating viable intracellular bacteria on THA. Intracellular survival assays were performed as described for phagocytosis, except that after 1 h of initial infection, cells were washed twice with PBS and antibiotic-containing medium was added to infected cells for an additional incubation time of up to 3 h. Infected cells were then washed three times, lysed with water, and the number of CFU was determined. Intracellular survival was expressed as the percentage of viable bacteria after 1 h of initial infection (time = 0).

### Dendritic Cell Activation and Pro-inflammatory Mediator Measurement

DCs were stimulated with the pre-opsonized *S. suis* strains (as described above) in culture medium (1 × 10^6^ CFU/mL; initial MOI = 1). In parallel experiments, cells were stimulated with 1, 10, or 100 μg/mL of rAgI/II. Supernatants were collected 16 h following stimulation, time at which secreted cytokine levels were maximal in the absence of *S. suis*-induced cytotoxicity as confirmed by measurement of release lactate dehydrogenase using the CytoTox96 Non-Radioactive Cytotoxicity Assay (Promega, Madison, WI, USA) (data not shown). Non-infected cells served as negative controls. Secreted levels of tumor necrosis factor (TNF), IL-1β, IL-6, IL-12p70, and CCL3 were quantified by sandwich ELISA using pair-matched antibodies from R&D Systems (Minneapolis, MN, USA) according to the manufacturer's recommendations.

### Whole Blood Bactericidal (Killing) Assay

Blood was collected from 6 to 10-week-old C57BL/6 mice and mixed with sodium heparin (Sigma-Aldrich). Leukocytes (9 × 10^6^ cells/mL on average) were transferred to a microtube containing 9 × 10^6^ CFU/mL of the *S. suis* strains (MOI = 1) and incubated for 4 h, mixing every 20 min. Assay conditions were chosen based on the kinetics of *S. suis* killing by murine blood (Auger et al., 2016). After incubation, cells were lysed by vortexing and appropriate dilutions plated on THA to determine viable bacterial counts. Resistance to bacterial killing by blood leukocytes was compared to incubation of the different strains in plasma only (obtained by centrifuging whole blood at 1,800 × *g* for 10 min at 4°C). Percentage of bacterial survival was determined using the following formula: (bacteria in blood/bacteria in plasma)/100%.

### Statistical Analyses

Normality of data was verified using the Shapiro-Wilk test. Accordingly, parametric (unpaired *t-*test) or non-parametric tests (Mann-Whitney rank sum test) were performed to evaluate statistical differences between groups. Log-rank (Mantel-Cox) tests were used to compare mouse survival. Each test was repeated in at least three independent experiments. *p* < 0.01 was considered as statistically significant.

## Results

### Antigen I/II Is Important for *S. suis* Serotype 9 Virulence and Development of Systemic Disease

**Figure 1:**
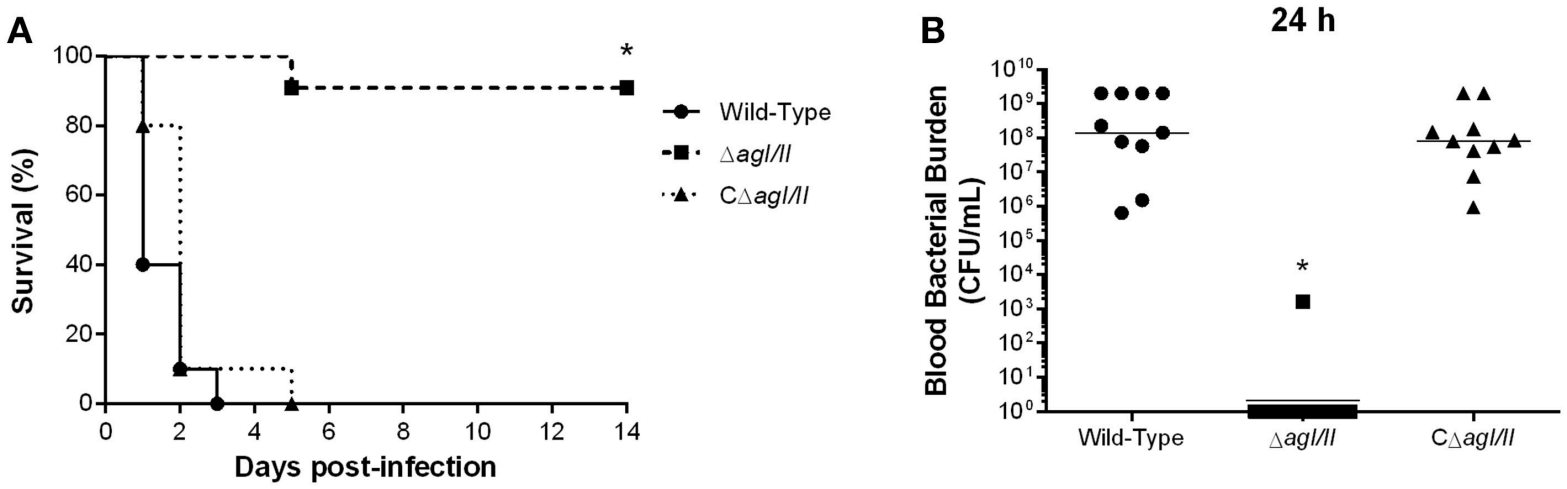
Presence of antigen I/II (AgI/II) is important for *Streptococcus suis* serotype 9 systemic virulence and blood persistence following intraperitoneal inoculation. Survival **(A)** and blood bacterial burden 24 h post-infection **(B)** of C57BL/6 mice following intraperitoneal inoculation of the *S. suis* serotype 9 wild-type, AgI/II-deficient mutant (Δ*agI/II*) or complemented (CΔ*agI/II*) strain. Data represent the survival curves **(A)** or geometric mean **(B)** of 10 mice/group. *p < 0.01 indicates a significant difference between survival or blood bacterial burden of mice infected with the wild-type strain and Δ*agI/II* mutant.

Unlike with *S. suis* serotype 2 (Berthelot-Herault et al., 2001; Dominguez-Punaro et al., 2007, 2008; Baums et al., 2009), experimental swine models of serotype 9 infection are very limited, which has greatly hindered pathogenesis studies. Using C57BL/6 mice, which are routinely used as a model for serotype 2 virulence studies (Dominguez-Punaro et al., 2008; Lachance et al., 2013; Auger et al., 2016), the role of AgI/II in *S. suis* serotype 9 systemic infection was evaluated following intraperitoneal inoculation. Mice infected with the wild-type strain developed clinical signs of systemic infection characterized by rough hair coat, swollen eyes, prostration, depression, and lethargy. These animals succumbed to infection within 3 days, with 60% of mortality 24 h post-infection (Figure 1A). By contrast, only 10% of mice infected with the Δ*agI/II* mutant succumbed to infection after 14 days (*p* < 0.001) (Figure 1A), and animals only presented transient signs of systemic infection (rough coat hair and swollen eyes) in the first 24 h of infection. Meanwhile, mice infected with the complemented strain, CΔ*agI/II*, presented similar clinical signs to those of mice infected with the wild-type strain, with 100% of mice succumbing to infection within the same time frame (Figure 1A). These results were confirmed in two subsequent infections (data not shown).

Since *S. suis* serotype 2 systemic infection is associated with persistent bacteremia (Auger et al., 2016), blood bacterial burden of mice infected with the serotype 9 wild-type, Δ*agI/II* or CΔ*agI/II* strain was evaluated. Twenty-four hours following infection, mice infected with the wild-type strain presented elevated blood bacterial burdens averaging 1 × 10^8^ CFU/mL (Figure 1B). Moreover, infection with the wild-type strain resulted in elevated bacterial burdens in the spleen and liver, suggesting systemic dissemination and persistence (data not shown). On the other hand, mice infected with the Δ*agI/II* mutant did not present measurable bacteremia 24 h post-infection (*p* < 0.001), with the exception of a single individual who eventually succumbed to infection 5 days post-infection (Figure 1B). Finally, and in accordance with survival, mice infected with the complemented strain, CΔ*agI/II*, presented similar blood bacterial burdens to those infected with the wild-type strain (Figure 1B).

**Figure 2:**
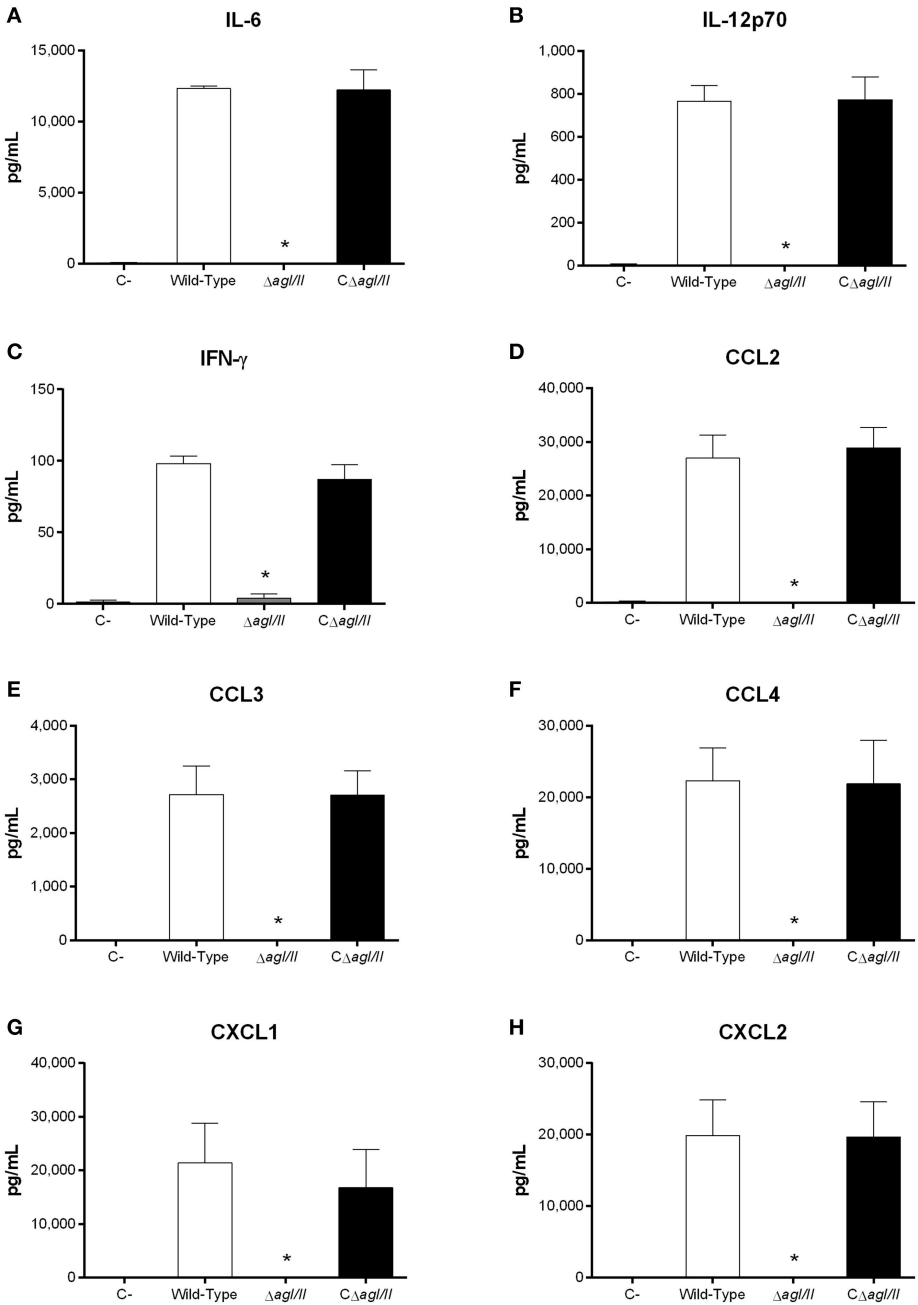
Reduced plasma pro-inflammatory mediator levels in the absence of antigen I/II (AgI/II) during *S. suis* serotype 9 systemic infection. Plasma levels of IL-6 **(A)**, IL-12p70 **(B)**, IFN-γ **(C)**, CCL2 **(D)**, CCL3 **(E)**, CCL4 **(F)**, CXCL1 **(G)**, and CXCL2 **(H)** in mice 12 h following following mock-infection or intraperitoneal inoculation of the *S. suis* serotype 9 wild-type, AgI/II-deficient mutant (Δ*agI/II*) or complemented (CΔ*agI/II*) strain. Data represent mean ± SEM (*n* = 8). C- denotes mock-infected mice. *p < 0.01 indicates a significant difference between plasma levels of mice infected with the wild-type strain and Δ*agI/II* mutant.

Exacerbated inflammation is a hallmark of the *S. suis* serotype 2-induced systemic infection and is responsible for host death due to sepsis and/or septic shock. In accordance, plasmatic levels of the different pro-inflammatory mediators evaluated (IL-6, IL-12p70, IFN-γ, CCL2, CCL3, CCL4, CXCL1, and CXCL2) were elevated in mice infected with the wild-type and CΔ*agI/II* strains (Figure 2). Meanwhile, not only were plasmatic levels of these mediators significantly lower in mice infected with the Δ*agI/II* mutant (*p* < 0.001), but they were similar to those of mock-infected mice (Figure 2).

### Antigen I/II Promotes *S. suis* Serotype 9 Resistance to Phagocytosis by Resident Peritoneal Macrophages

**Figure 3:**
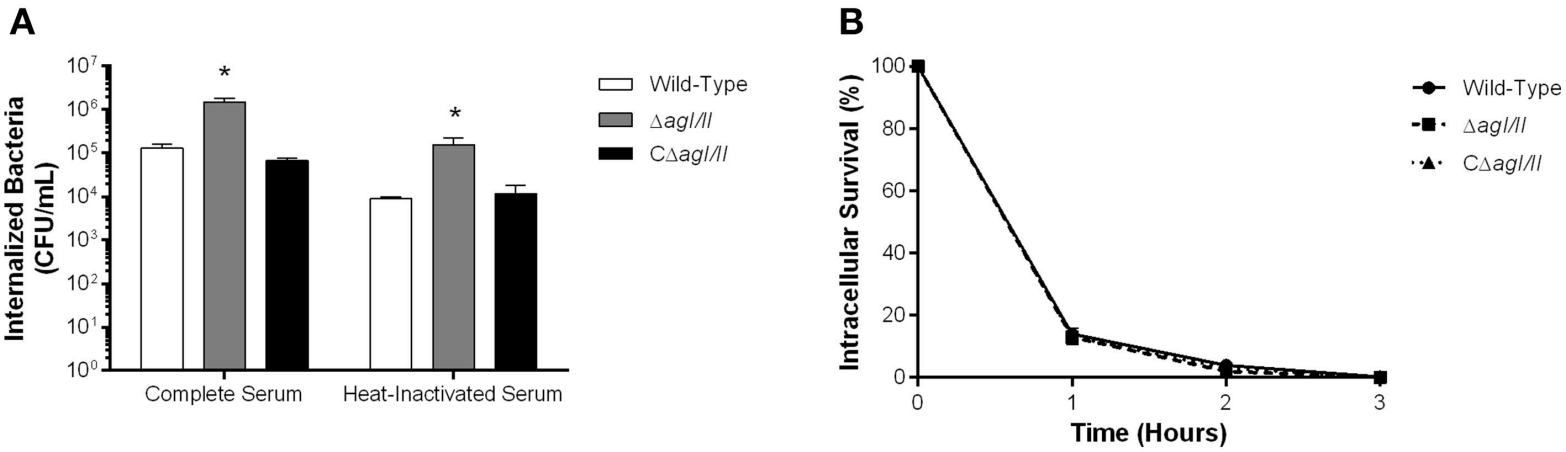
Presence of antigen I/II (AgI/II) promotes *S. suis* serotype 9 resistance to phagocytosis by resident peritoneal macrophages but not intracellular survival. **(A)** Phagocytosis of the *S. suis* serotype 9 wild-type, AgI/II-deficient mutant (Δ*agI/II*) or complemented (CΔ*agI/II*) strains pre-opsonized with either 20% complete or heat-inactivated normal mouse serum by resident peritoneal macrophages following 1 h of infection. **(B)** Intracellular survival kinetics of the wild-type, Δ*agI/II*, and CΔ*agI/II* strains pre-opsonized with 20% complete normal mouse serum within resident peritoneal macrophages following antibiotic treatment. Data represent the mean ± SEM (*n* = 4). *p < 0.01 indicates a significant difference between the wild-type strain and Δ*agI/II* mutant.

The compromised blood presence/persistence of *S. suis* serotype 9 in the absence of AgI/II following intraperitoneal inoculation suggested that AgI/II might be implicated in the interactions with peritoneal macrophages, which are the first innate immune cells encountered following intraperitoneal inoculation. Consequently, the role of *S. suis* serotype 9 AgI/II in resistance to phagocytosis by resident peritoneal macrophages was evaluated by infecting cells with bacteria pre-opsonized with either 20% complete or heat-inactivated normal mouse serum, used to evaluate the role of complement. After 1 h of incubation, 1 × 10^5^ CFU of the wild-type strain pre-opsonized with complete serum were internalized, representing 1% of inoculum (Figure 3A). Meanwhile the Δ*agI/II* mutant was significantly more internalized (*p* < 0.01), with approximately 10% of bacteria recovered (Figure 3A). On the other hand, complementation (CΔ*agI/II*) restored wild-type phenotype (Figure 3A). Interestingly, pre-opsonization with heat-inactivated mouse serum significantly reduced phagocytosis of all strains by approximately 10-fold (*p* < 0.01) (Figure 3A), although the deficient and complemented mutants were still more and equally internalized, respectively, than the wild type-strain (Figure 3A).

Unlike with phagocytosis, AgI/II did not modulate intracellular survival of *S. suis* serotype 9 pre-opsonized with either complete serum (Figure 3B) or heat-inactivated serum (data not shown). Indeed, only 15% of initially internalized wild-type, Δ*agI/II* or CΔ*agI/II* bacteria were viable after 1 h of antibiotic treatment, with <5% remaining after 2 h of antibiotic treatment, and no viable bacteria recovered after 3 h of antibiotic treatment (Figure 3B). Taken together, these results suggest that while AgI/II affects *S. suis* phagocytosis by promoting resistance of *S. suis* serotype 9 to resident peritoneal macrophages, it does not promote intracellular bacterial survival, nor does it interfere with complement deposition.

### Antigen I/II Promotes *S. suis* Serotype 9 Resistance to Killing by Whole Blood

**Figure 4:**
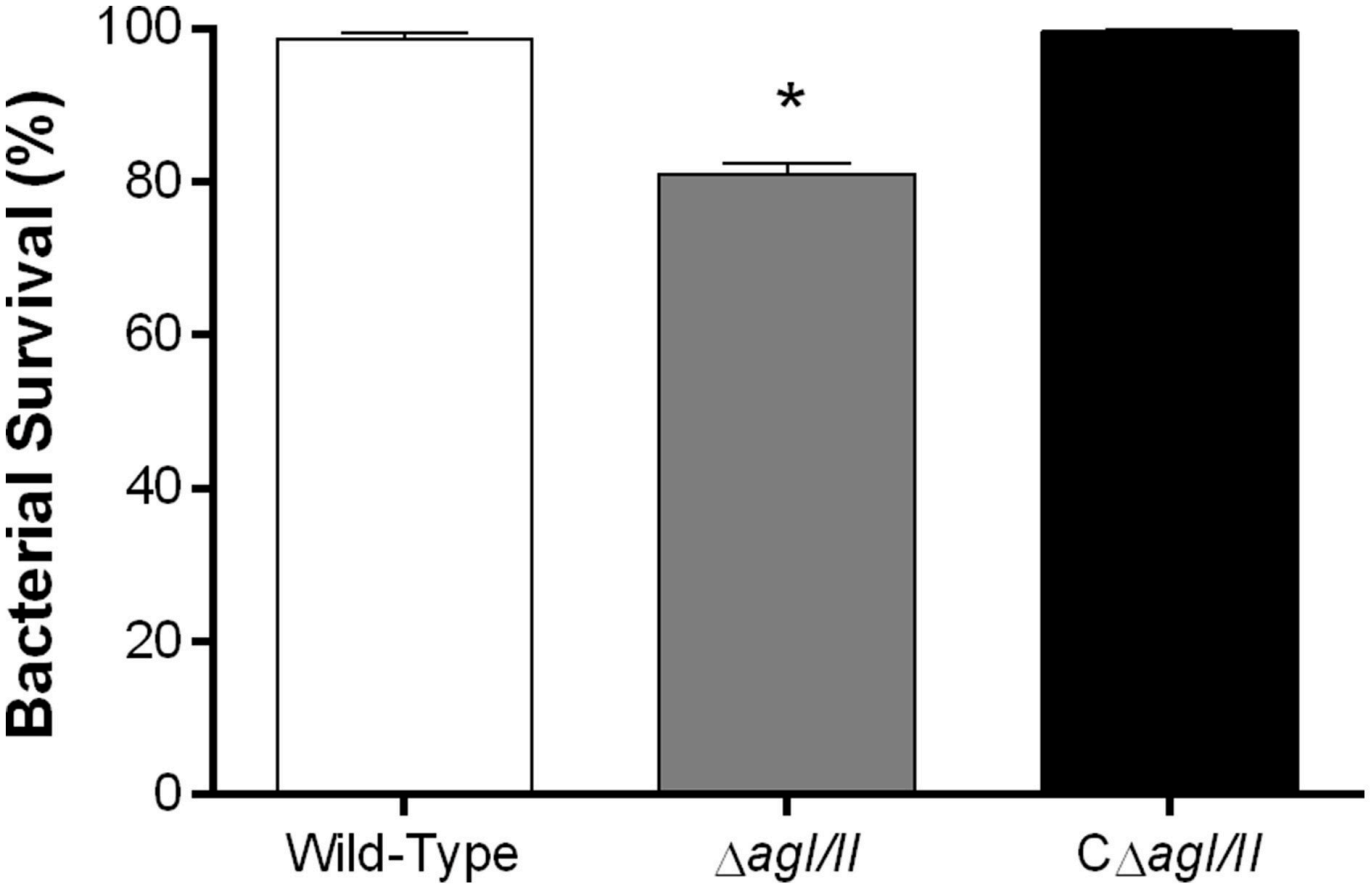
Presence of antigen I/II (AgI/II) promotes *S. suis* serotype 9 resistance to killing by whole blood. Resistance of wild-type, AgI/II-deficient mutant (Δ*agI/II*) or complemented (CΔ*agI/II*) strains to the bactericidal effect of murine whole blood after 4 h of incubation. Percentage of bacterial survival was calculated in comparison to bacteria in plasma alone. Data represent the mean ± SEM (*n* = 4). *p < 0.01 indicates a significant difference between the wild-type strain and Δ*agI/II* mutant.

From the peritoneal cavity, non-internalized bacteria will rapidly reach the bloodstream, where resistance to killing by leukocytes is required for persistence and systemic dissemination. Consequently, the role of AgI/II in resistance of *S. suis* serotype 9 to killing by mouse whole blood was evaluated. The wild-type strain was almost completely resistant to killing by blood, with 99% of survival after 2 h (results not shown) and 4 h of incubation (Figure 4). By contrast, the Δ*agI/II* mutant was significantly less resistant to killing (*p* < 0.001), with 80% of survival (Figure 4). Meanwhile, complementation (CΔ*agI/II*) restored wild-type phenotype (Figure 4).

### Antigen I/II Modulates the Interactions of *S. suis* Serotype 9 With Dendritic Cells: Role in Phagocytosis Resistance, Intracellular Survival, and Pro-inflammatory Mediator Induction

**Figure 5:**
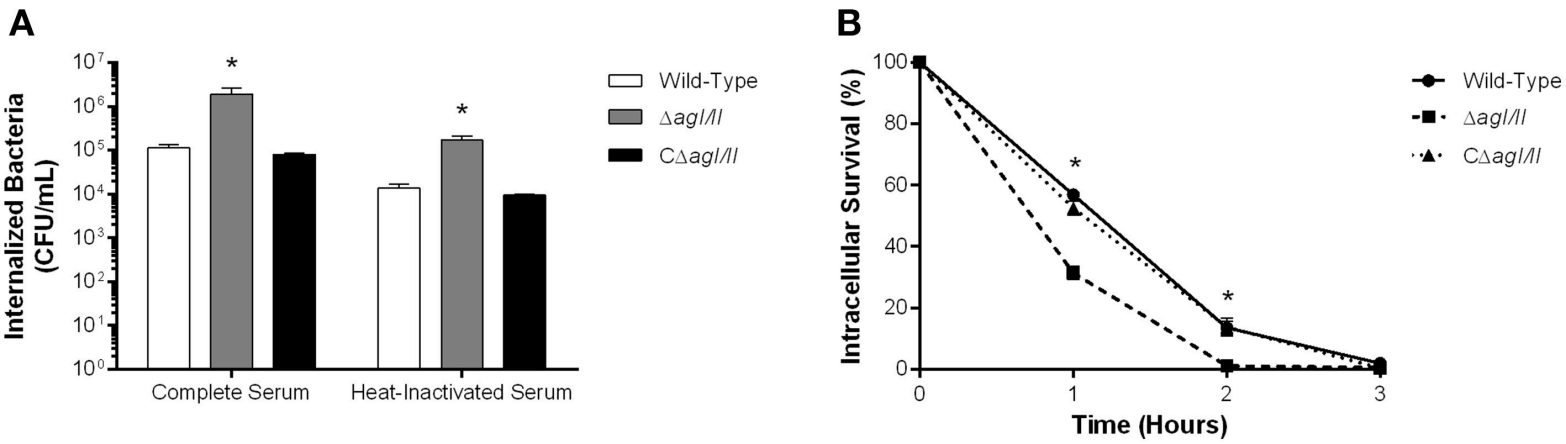
Presence of antigen I/II (AgI/II) promotes *S. suis* serotype 9 resistance to phagocytosis by dendritic cells (DCs) and intracellular survival. **(A)** Phagocytosis of the *S. suis* serotype 9 wild-type, AgI/II-deficient mutant (Δ*agI/II*) or complemented (CΔ*agI/II*) strains pre-opsonized with either 20% complete or heat-inactivated normal mouse serum by DCs following 1 h of infection. **(B)** Intracellular survival kinetics of the wild-type, Δ*agI/II*, and CΔ*agI/II* strains pre-opsonized with 20% complete normal mouse serum within DCs following antibiotic treatment. Data represent the mean ± SEM (*n* = 4). *p < 0.01 indicates a significant difference between the wild-type strain and Δ*agI/II* mutant.

As mentioned above, persistent bacteremia results in systemic dissemination of *S. suis*, with elevated bacterial burdens recovered in various internal organ following infection with serotype 2 (Dominguez-Punaro et al., 2007). In these organs, *S. suis* will encounter different resident innate immune cells, including DCs. Indeed, since the importance of the interactions between *S. suis* serotype 2 and dendritic cells has been demonstrated in the past (Lecours et al., 2011, 2012; Auger et al., 2017, 2018), these interactions could also be important for serotype 9. As such, the role of AgI/II in the interactions with DCs was evaluated. After 1 h of incubation, 1 × 10^5^ CFU of the *S. suis* serotype 9 wild-type strain pre-opsonized with complete serum was internalized by DCs, corresponding to 0.1% of inoculum (Figure 5A). However, the Δ*agI/II* mutant was significantly more internalized (*p* < 0.01), with 1 × 10^6^ CFU recovered, equivalent to 1% of inoculum, while complementation (CΔ*agI/II*) restored wild-type levels (Figure 5A). Similarly to what was observed with macrophages, pre-opsonization with heat-inactivated serum significantly reduced DC phagocytosis of all strains by 10-fold (*p* < 0.01) (Figure 5A). Once again, however, the Δ*agI/II* mutant was significantly more internalized by DCs (*p* < 0.01) (Figure 5A).

Survival of internalized wild-type bacteria was initially elevated, with 60% of viability after 1 h of antibiotic treatment (Figure 5B). However, viability quickly decreased thereafter, with 15 and 0% after 2 and 3 h of antibiotic treatment, respectively (Figure 5B). By contrast, intracellular survival of the Δ*agI/II* mutant was significantly reduced (*p* < 0.01), with only 30% after 1 h of antibiotic treatment, before reaching 0% after 2 h of antibiotic treatment (Figure 5B). Importantly, complementation (CΔ*agI/II***)** restored wild-type phenotype (Figure 5B). Similar results were also obtained using heat-inactivated serum (data not shown).

**Figure 6:**
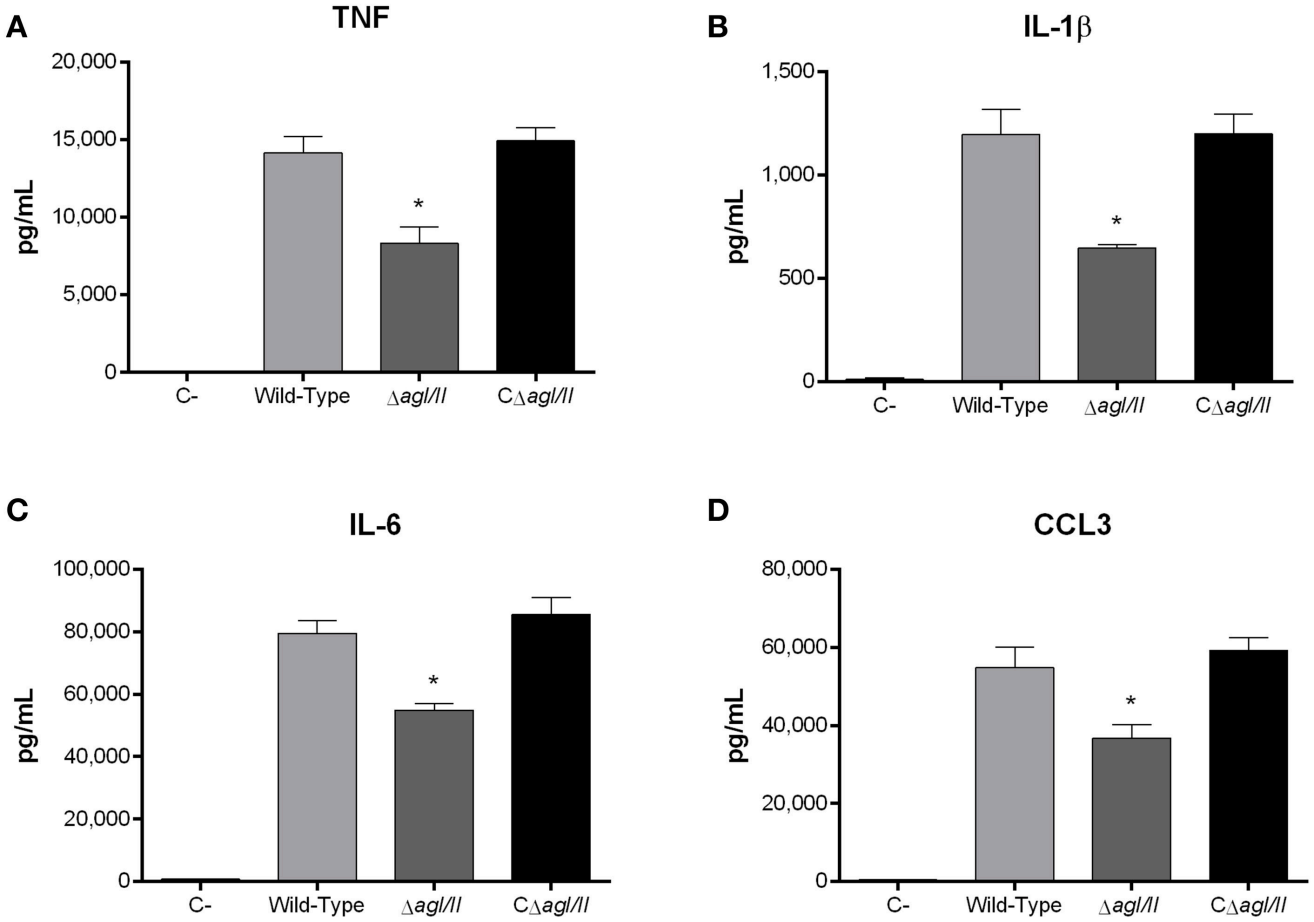
Presence of antigen I/II (AgI/II) modulates *S. suis* serotype 9-induced dendritic cell (DC) pro-inflammatory mediator production. Pro-inflammatory mediator production by DCs following 16 h of infection with the *S. suis* serotype 9 wild-type, AgI/II-deficient mutant (Δ*agI/II*) or complemented (CΔ*agI/II*) strains pre-opsonized with 20% complete normal mouse serum, as measured by ELISA. Production of TNF **(A)**, IL-1β **(B)**, IL-6 **(C)**, and CCL3 **(D)**. Data represent the mean ± SEM (*n* = 4). C- denotes cells in medium alone. *p < 0.01 indicates a significant difference between the wild-type strain and Δ*agI/II* mutant.

**Figure 7:**
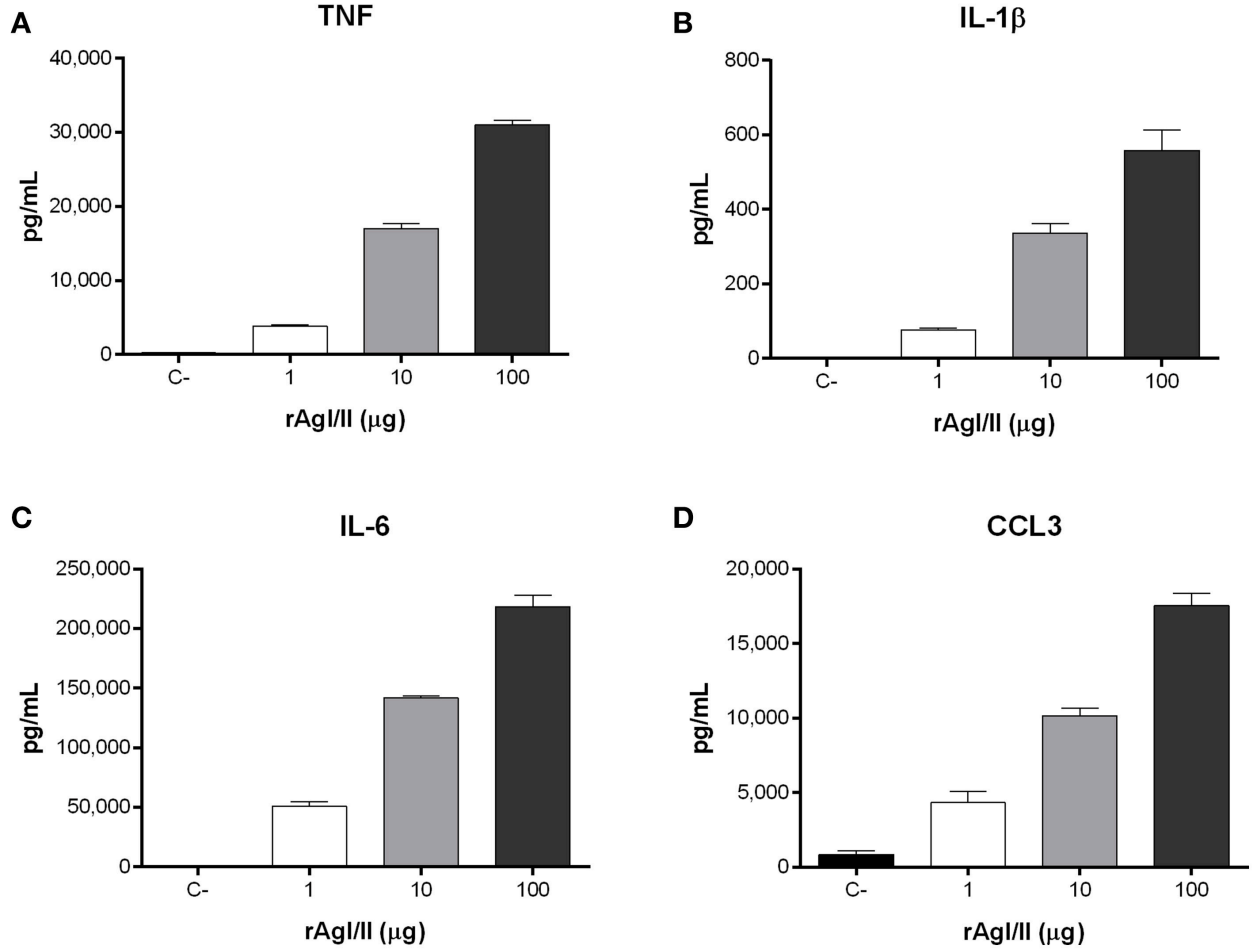
*Streptococcus suis* serotype 9 antigen I/II (rAgI/II) induces an important pro-inflammatory response from dendritic cells (DCs). Pro-inflammatory mediator production by DCs following 16 h of stimulation with 1, 10, or 100 μg/mL of recombinant AgI/II (rAgI/II), as measured by ELISA. Production of TNF **(A)**, IL-1β **(B)**, IL-6 **(C)**, and CCL3 **(D)**. Data represent the mean ± SEM (*n* = 3). C- denotes cells in medium alone.

Recognition of *S. suis* by DCs not only activates phagocytic mechanisms involved in bacterial clearance, but it also induces pro-inflammatory mediator production (Lecours et al., 2011). The *S. suis* serotype 9 wild-type strain induced important levels of TNF, IL-1β, IL-6, and CCL3 from DCs (Figure 6), but barely detectable levels of IL-12p70 (data not shown). Pro-inflammatory mediator production by DCs was significantly reduced following infection with the Δ*agI/II* mutant by 30–50% depending on the mediator (*p* < 0.01) (Figure 6). Meanwhile, complementation (CΔ*agI/II*) restored wild-type phenotype, suggesting that the *S. suis* serotype 9 AgI/II possesses important immunostimulatory properties (Figure 6). Consequently, DCs were stimulated with different concentrations of rAgI/II (1, 10, or 100 μg/mL). In accordance, rAgI/II induced elevated levels of TNF, IL-1β, IL-6, and CCL3 from DCs in a dose-dependent manner (Figure 7). Similar results were also obtained with rAgI/II pretreated with 20 μg/mL of polymyxin B sulfate, confirming that results obtained are not due to lipopolysaccharide contamination (data not shown). Moreover, endotoxin levels were quantified using the Limulus Amebocyte Lysate Assay and determined to be below the detection level of 0.06 EU/mL (data no shown).

**Figure 8:**
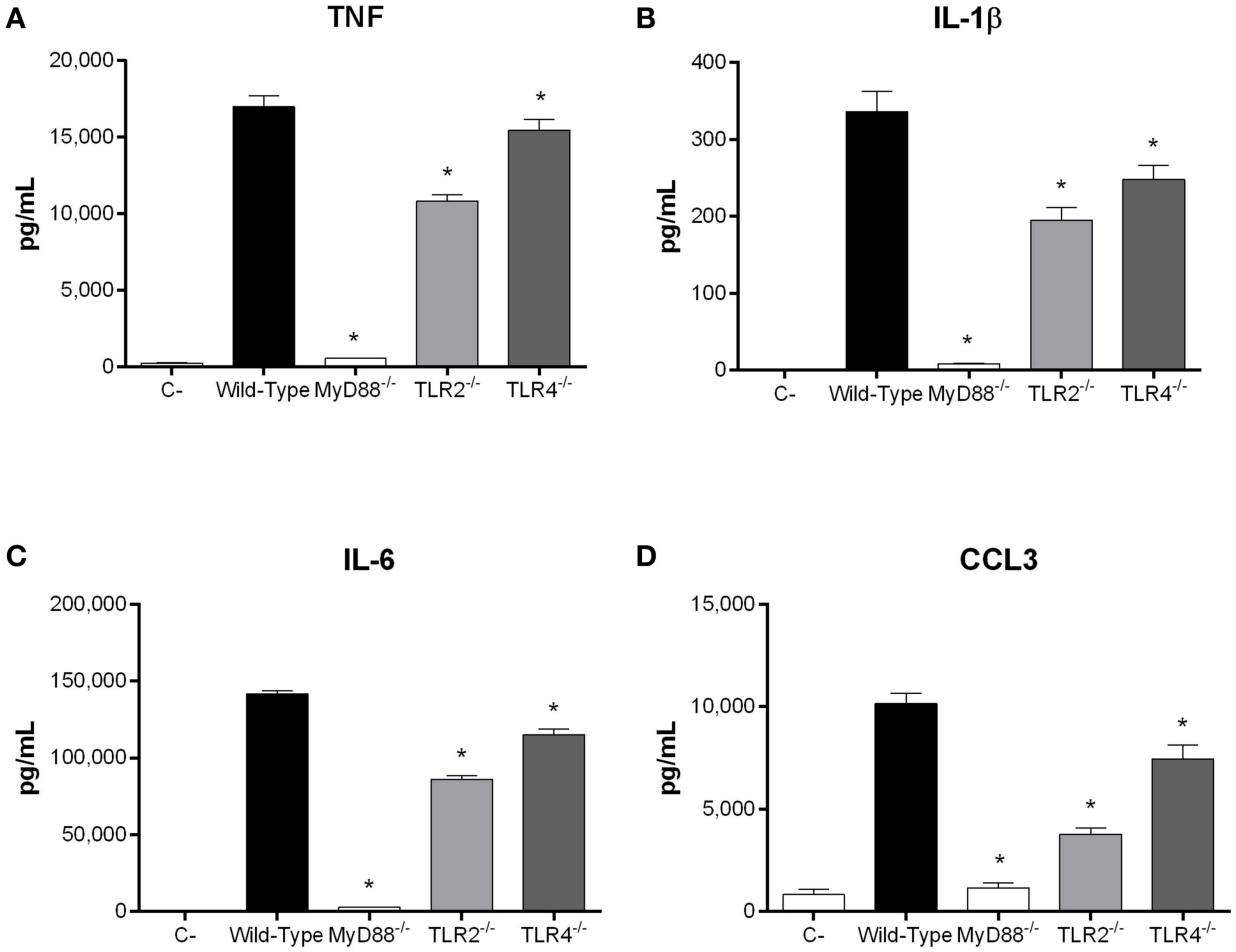
Recognition of *S. suis* serotype 9 antigen I/II (AgI/II) by dendritic cells (DCs) requires MyD88-dependent signaling. Pro-inflammatory mediator production by wild-type, MyD88^−/−^, Toll-like receptor (TLR) 2^−/−^ or TLR4^−/−^ DCs following 16 h of stimulation with 10 μg/mL of recombinant AgI/II, as measured by ELISA. Production of TNF **(A)**, IL-1β **(B)**, IL-6 **(C)**, and CCL3 **(D)**. Data represent the mean ± SEM (*n* = 3). C- denotes cells in medium alone. *p < 0.01 indicates a significant difference between wild-type and MyD88^−/−^, TLR2^−/−^, or TLR4^−/−^ DCs.

Recognition of *S. suis* by DCs mainly involves the membrane-associated Toll-like receptor (TLR) pathway, in which the adaptor protein MyD88 is central to signal transduction (Fittipaldi et al., 2012; Segura et al., 2016). Moreover, being an extracellular pathogen, recognition of *S. suis* has been mostly associated with surface TLRs, where TLR2 plays a predominant role (Graveline et al., 2007; Wichgers Schreur et al., 2010; Lecours et al., 2012). Though TLR4 is classically associated with recognition of Gram-negative bacteria, it was also suggested to recognize the *S. suis* secreted cytolysin suilysin (Bi et al., 2015). As such, the role of the TLR pathway in rAgI/II recognition by DCs was evaluated using MyD88^−/−^ cells. In the absence of MyD88, production of TNF, IL-1β, IL-6, and CCL3 following stimulation with 10 μg/mL of rAgI/II was almost completely abolished (*p* < 0.001) (Figure 8). Meanwhile, absence of TLR2 resulted in a significant but partial decrease of TNF, IL-1β, and IL-6 production (*p* < 0.01), equivalent to approximately 60% of wild-type DC production, with a greater implication in CCL3 production (*p* < 0.001) (Figure 8). Surprisingly, this was also the case for TLR4, absence of which resulted in a significant decrease of IL-1β, IL-6, and CCL3 (*p* < 0.01), but not of TNF production (Figure 8). However, this decrease was generally less than in the absence of TLR2 (Figure 8). Similar results were also obtained using 1 or 100 μg/mL (data not shown). Taken together, these results demonstrate that the *S. suis* serotype 9 AgI/II induces a pro-inflammatory response from DCs following its recognition by TLRs, with TLR2 playing an important role.

### Intravenous Inoculation of *S. suis* Serotype 9 Confirms the Role of Antigen I/II in Virulence and Development of Systemic Disease

**Figure 9:**
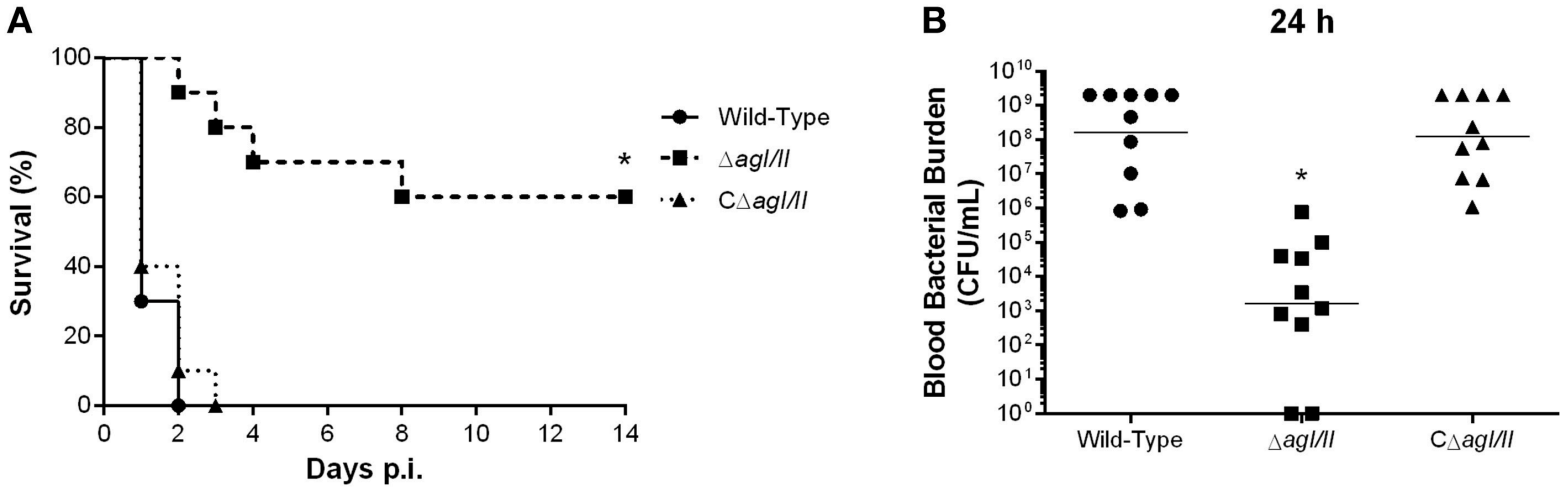
Presence of antigen I/II (AgI/II) participates in *S. suis* serotype 9 systemic virulence and blood persistence following intravenous inoculation. Survival **(A)** and blood bacterial burden 24 h post-infection **(B)** of C57BL/6 mice following intravenous inoculation of the *S. suis* serotype 9 wild-type, AgI/II-deficient mutant (Δ*agI/II*) or complemented (CΔ*agI/II*) strain. Data represent the survival curves **(A)** or geometric mean **(B)** of 10 mice/group. *p < 0.01 indicates a significant difference between survival or blood bacterial burden of mice infected with the wild-type strain and Δ*agI/II* mutant.

To study if the interactions with local macrophages in the peritoneal cavity might have influenced the virulence pattern observed, the role of AgI/II was also studied with mice infected by intravenous injection. Inoculation of the *S. suis* serotype 9 wild-type strain resulted in the development of systemic clinical disease similar to following intraperitoneal inoculation, with 100% of mice succumbing to infection within 2 days (Figure 9A). Meanwhile, mice infected with the Δ*agI/II* mutant succumbed significantly less to infection (*p* < 0.01), with 40% of mortality after 14 days (Figure 9A). Though clinical signs were less severe in Δ*agI/II*-infected mice, most mice presented some signs of systemic infection such as rough coat hair, swollen eyes, prostration, and depression, which persisted for at least 48 h (data not shown). By contrast, the complemented strain, CΔ*agI/II*, caused similar mortality to the wild-type strain (Figure 9A).

In accordance with mortality, blood bacterial burden of mice infected with the wild-type strain was elevated at 24 h post-infection, averaging 1 × 10^8^ CFU/mL, while that of mice infected with the Δ*agI/II* mutant was significantly lower (*p* < 0.001) (Figure 9B). Unlike following intraperitoneal inoculation, however, mice infected with the Δ*agI/II* mutant presented detectable blood bacterial burdens, which averaged 1 × 10^3^ CFU/mL (Figure 9B). Finally, blood bacterial burden of mice infected with the complemented strain was similar to that of wild-type strain-infected mice (Figure 9B).

## Discussion

*Streptococcus suis* serotype 9 has emerged as one of the most frequently isolated serotypes in recent years, yet little information is available regarding its pathogenesis. Though we recently demonstrated that AgI/II is required for the initial steps of the *S. suis* serotype 9 pathogenesis (Chuzeville et al., 2017), its role in the systemic infection, including interactions with phagocytes, remained unknown.

Well-standardized experimental animal models of *S. suis* infection remain limited and were mostly described for serotype 2 (Beineke et al., 2008; Segura et al., 2017). Though successful experimental infections have been carried out in pigs, most have used cesarean-derived colostrum-deprived animals, which are germ-free. This model greatly differs from “conventional” pigs, which are colonized by *S. suis*, rendering them more resistant to infection (Dekker et al., 2012, 2013, 2017). Evaluation of virulence of serotype 9 strains in pigs is even more complicated than that of serotype 2 (Beineke et al., 2008) Moreover, it is important to note that Canadian serotype 9 strains (including that used in the present study) present a lower virulence profile than European strains (Zheng et al., 2018). Indeed, we were previously unable to induce disease in an intranasal porcine model of infection with the 1135776 strain used herein (Chuzeville et al., 2017).

Due to these constraints, a C57BL/6 mouse model of *S. suis* serotype 9 systemic infection was developed using the intraperitoneal and intravenous route of inoculation. Mice presented similar clinical signs of systemic disease characteristic of sepsis and septic shock to those previously described for serotype 2 (Dominguez-Punaro et al., 2008; Auger et al., 2016), and this using both inoculation routes. Furthermore, serotype 9-induced systemic disease was characterized by elevated and persistent blood bacterial burden and a strong host systemic inflammatory response composed of a variety of pro-inflammatory mediators, resulting in host death. As such, these infection models could be useful for future *S. suis* serotype 9 pathogenesis studies.

We demonstrated an important role of AgI/II in systemic infection and development of clinical disease during *S. suis* serotype 9 infection. Interestingly, host survival following infection with the Δ*agI/II* mutant differed between routes of inoculation. Though different immune cells reside in the peritoneal cavity, macrophages are the main innate immune cell type (Ray and Dittel, 2010). After intraperitoneal inoculation of the Δ*agI/II* mutant, local cells are probably responsible for partial bacterial elimination. In fact, the lack of AgI/II increased internalization by macrophages (with rapid intracellular killing), demonstrating that the *S. suis* serotype 9 AgI/II affects *S. suis* phagocytosis. Data from the present work cannot dissect between a direct effect of AgI/II on *S. suis* phagocytosis alone and an indirect effect via modulation of other host cell functions, with consequent effects on the general phagocytic capacity of the cells, as previously described for the *S. suis* serotype 2 capsular polysaccharide (Segura et al., 2004; Houde et al., 2012). Future studies using, for example, rAgI/II linked to beads, will be necessary to further study the mechanisms involved. Alongside, participation of AgI/II in *S. suis* self-aggregation might reduce the number of bacteria activating phagocytic mechanisms (Chuzeville et al., 2017), as was previously reported for neutrophils (Galdiero et al., 1988; Champion et al., 2008). In addition to macrophages, a certain role of neutrophils recruited to the peritoneal cavity following inoculation cannot be excluded (Czuprynski and Brown, 1987). However, neutrophil infiltration is not instant, peaking between 3 and 6 h post-infection following inoculation of GBS (Biondo et al., 2014). If bacteria are not eliminated locally and reach the bloodstream, animals will succumb to infection. Indeed, the single animal infected with the Δ*agI/II* mutant presenting a significant blood bacterial burden died 5 days post-infection. When injected directly into the bloodstream, the Δ*agI/II* mutant caused some clinical signs, a measurable bacteremia, and 40% of mortality, suggesting a partial role of this protein in bacterial survival once in the bloodstream. Indeed, AgI/II was involved in bacterial survival using an *in vitro* whole blood killing assay. Taken together, these results indicate an implication of additional bacterial factors, which will need to be identified in future studies.

Once in blood, presence of sufficiently elevated burdens will allow *S. suis* to colonize different internal organs during the systemic phase of the infection (Dominguez-Punaro et al., 2007, 2008). DCs are important tissue-resident phagocytes central to the *S. suis* serotype 2 pathogenesis and are involved in innate immune functions such as phagocytosis and pro-inflammatory mediator production (Lecours et al., 2011, 2012; Auger et al., 2017, 2018). As observed with macrophages, *S. suis* serotype 9 AgI/II confers partial bacterial protection against phagocytosis by DCs. Notably, the phagocytic capacities of DCs toward *S. suis* serotype 9 were markedly less than those of peritoneal macrophages. Though macrophages and DCs are both professional phagocytes, it was previously demonstrated that macrophages exhibit more pronounced phagocytic activities toward *Staphylococcus aureus* and *Escherichia coli* than do DCs (Nagl et al., 2002). Moreover, macrophages are better at killing ingested bacteria than DCs (Nagl et al., 2002). Similar results were obtained herein with *S. suis* serotype 9: DCs are less efficient at killing intracellular *S. suis* serotype 9 than macrophages, with 60 and 15 % intracellular survival, respectively. In fact, this difference in killing efficiency might also explain why AgI/II participated in intracellular bacterial survival in DCs but not macrophages, since the phagosomal environment faced by *S. suis* in DCs is less harsh than that in macrophages (Savina and Amigorena, 2007). Indeed, we previously demonstrated that though AgI/II promotes bacterial survival at different pH by participating in acid stress, its effect is less marked as the pH lowers (Chuzeville et al., 2017).

It is interesting to compare the role of the *S. suis* serotype 9 AgI/II with that of AspA of GAS. AspA was also reported to play a role in the resistance to phagocytic killing by murine macrophages and human neutrophils (Franklin et al., 2013). This resemblance between AgI/II and AspA, regardless of sharing only 35% amino acid sequence homology (Chuzeville et al., 2017), suggests it might be a conserved property of AgI/II family members. However, persistence in blood was AspA-independent, indicating that while it confers anti-phagocytic properties *in vitro*, this is not necessarily the case *in vivo* (Franklin et al., 2013). By contrast, AgI/II was important for persistence of *S. suis* serotype 9 in blood and resistance to its bactericidal effect, making this the first time that an AgI/II family member is described to be involved in bacterial survival and persistence in blood.

Being an extracellular pathogen, *S. suis* has developed tools to inhibit phagocytosis and killing, including interference in complement deposition at the bacterial surface since this promotes internalization via opsonophagocytosis (Chabot-Roy et al., 2006; Lecours et al., 2011). Indeed, *S. suis* serotype 2 suilysin and cell wall modifications (Lecours et al., 2011), as well as the factor H-binding protein (Roy et al., 2016), are involved in this interference. However, while AgI/II affects *S. suis* serotype 9 phagocytosis, this role was complement-independent. Interestingly, unlike serotype 2 (Chabot-Roy et al., 2006; Lecours et al., 2011), the serotype 9 1135776 strain was relatively sensitive to opsonophagocytosis, with presence of complement increasing its internalization by macrophages and DCs by 10-fold. This may indicate differential expression of factors involved in the interference of complement deposition. Amongst these, sialic acid, present in the serotype 2 capsular polysaccharide but absent from that of serotype 9, might be involved (Van Calsteren et al., 2010; Vinogradov et al., 2016). Though the role of sialic acid in the *S. suis* pathogenesis has not been clearly defined, presence of sialic acid in GBS modulates neutrophil functions and virulence (Weiman et al., 2010). Furthermore, the serotype 9 CPS uniquely contains a labile 4-keto sugar (2-acetamido-2,6-dideoxy-β-D-xylo-hexopyranos-4-ulose), whose role also remains unknown (Vinogradov et al., 2016). However, future studies using other serotype 9 strains in comparison with serotype 2 will be needed to confirm these results.

Though serotype 9 is responsible for invasive disease resulting in exacerbated inflammatory responses, including sudden death (Goyette-Desjardins et al., 2014), the inflammatory response induced by this serotype remains relatively unknown. The low plasmatic inflammatory markers in mice infected with the Δ*agI/II* mutant was most probably due to the absence of severe clinical signs following rapid clearance from the systemic compartment, thus resulting in little immune activation. However, results obtained herein demonstrate that AgI/II itself partially modulates the induction of pro-inflammatory mediators. In fact, it possesses important immunostimulatory properties responsible for DC activation and induction of a pro-inflammatory response. A similar role was also observed for SspA and SspB of *S. gordonii*, which participate in the induction of several mediators from murine DCs and from human lung epithelial cells (Andrian et al., 2012). Furthermore, we demonstrated that recognition of AgI/II was MyD88-dependent, and partially TLR2- and TLR4-dependent. These results suggest that other MyD88-dependent TLRs are also involved in the recognition of AgI/II, or a synergistic role of TLR2 and TLR4. To our knowledge, this is the first study to demonstrate a role of the TLR pathway in AgI/II family member recognition. In fact, practically no information is available regarding the immunostimulatory properties of these proteins, including the cellular receptors and pathways. Though the role of MyD88 signaling, including TLR2 and TLR4, remained unknown *in vivo*, we recently demonstrated for serotype 2 that absence of MyD88 results in rapid mouse death due to lack of inflammation and uncontrolled bacterial burden, while absence of TLR2 or TLR4 had only minor effects (Auger et al., 2019). As such, *in vivo* data obtained with MyD88^−/−^ mice are similar to *in vitro*, while this is not the case for TLR2 nor TLR4. In fact, our results suggest that compensation and/or synergism occurs *in vivo* regarding TLR2 or TLR4 individually (Auger et al., 2019). Although no study using serotype 9 strains has yet been conducted, similar results were obtained with genotypically and phenotypically distinct serotype 2 strains, suggesting that MyD88 signaling is dependent on recognition of conserved *S. suis* motifs (Auger et al., 2019). Consequently, this may also be the case for serotype 9, but future studies will be necessary to confirm this.

In conclusion, *S. suis* serotype 9 causes a systemic infection resulting in the development of clinical disease and host death. As with serotype 2, this infection is characterized by exacerbated inflammation induced by an uncontrolled and persistent bacterial presence in the systemic compartment. Not only does presence of AgI/II affect *S. suis* phagocytosis by promoting resistance to phagocytic cells, but it also participates in innate immune cell activation, and by consequence, inflammation. As such, *S. suis* serotype 9 AgI/II is an important factor involved in not only the initial steps of its pathogenesis in pigs, but also for virulence during systemic infection and development of disease in a mouse model. In fact, this is the first study to describe a role of an AgI/II family member in systemic bacterial disease.

## Ethics Statement

This study was carried out in accordance with the recommendations of the guidelines and policies of the Canadian Council on Animal Care and the principles set forth in the Guide for the Care and Use of Laboratory Animals. The protocols and procedures were approved by the Animal Welfare Committee of the University of Montreal (permit number Rech-1570).

## Author Contributions

J-PA, MS, and MG conceived and designed the experiments. J-PA and A-CB performed the experiments. J-PA and MG analyzed the data. J-PA, MS, and MG contributed to the writing of the manuscript. All authors have read and approved the manuscript.

### Conflict of Interest Statement

The authors declare that the research was conducted in the absence of any commercial or financial relationships that could be construed as a potential conflict of interest.

## Abbreviations

AgI/II: antigen I/II
CFU: colony forming unit
CCL: C-C motif chemokine ligand
CXCL: C-X-C motif chemokine ligand
DC: dendritic cells
GAS: Group A *Streptococcus*
GBS: Group B *Streptococcus*
IFN: interferon
IL: interleukin
MOI: multiplicity of infection
PBS: phosphate-buffered saline
rAgI/II: recombinant AgI/II
SEM: standard error of the mean
THA: THB agar
THB: Todd Hewitt broth
TLR: Toll-like receptor
TNF: tumor necrosis factor.
